# Effectivity of near-peer teaching in training of basic surgical skills – a randomized controlled trial

**DOI:** 10.1186/s12909-021-02590-2

**Published:** 2021-03-12

**Authors:** Zsolt Pintér, Dániel Kardos, Péter Varga, Eszter Kopjár, Anna Kovács, Péter Than, Szilárd Rendeki, László Czopf, Zsuzsanna Füzesi, Ádám Tibor Schlégl

**Affiliations:** 1 Medical Simulation Education Centre, University of Pécs, Medical School, Pécs, Hungary; 2 Department of Paediatrics, University of Pécs, Medical School, Pécs, Hungary; 3 Department of Primary Health Care, University of Pécs, Medical School, Pécs, Hungary; 4 Department of Orthopaedics, University of Pécs, Medical School, Akác street 1., Pécs, H-7632 Hungary; 5 Department of Operational Medicine, University of Pécs, Medical School, Pécs, Hungary; 6 1st Department of Medicine, University of Pécs, Medical School, Pécs, Hungary; 7 Dean’s Office, University of Pécs, Medical School, Pécs, Hungary; 8 Department of Behavioural Sciences, University of Pécs, Medical School, Pécs, Hungary

**Keywords:** Near-peer teaching, Surgical education, RCT, Basic surgical skills, Suturing

## Abstract

**Background:**

Near-peer teaching (NPT) is a special way of teaching where the tutor is one or more academic years ahead of the person being tutored. The literature agrees on the benefits of the method, but there are only a few publications examining its effectiveness using objective methods. The aim of our study was to examine the effectiveness of NPT in the training of basic surgical skills.

**Methods:**

We included 60 volunteer students who participated in a 20 × 45 min long surgical skills course. Based on the results of a pre-course test, we randomly divided the students into six equal groups. All groups completed the same curriculum, with three groups being assisted by a NPT tutor. After the course, they completed the same test as at the beginning. The exams were recorded on anonymized videos and were blindly evaluated. The students’ satisfaction was monitored using a self-administered online anonymous questionnaire. Statistical analysis was performed using the Mann-Whitney and Wilcoxon tests.

**Results:**

Overall, student performance improved with completion of the course (from 119.86 to 153.55 points, *p* <  0.01). In groups where a NPT tutor assisted, students achieved a significantly better score (37.20 vs. 30.18 points improvement, *p* = 0.036). The difference was prominent in surgical knotting tasks (14.73 vs. 9.30 points improvement, *p* <  0.01). In cases of suturing (15.90 vs. 15.46 points) and laparoscopy (7.00 vs. 4.98 points), the presence of the NPT tutor did not significantly affect development. Based on student feedback, although students positively assessed the presence of NPT, it did not significantly improve students’ overall satisfaction since it was already 4,82 on a scale of 5 in the control group.

**Conclusions:**

Overall, involving a NPT tutor had a positive impact on student development. An outstanding difference was observed in connection with knotting techniques.

## Background

Near-peer teaching (NPT) is gaining popularity as an effective teaching method in medicine, especially in anatomy and basic sciences. Furthermore, its benefits have already been documented in detail in surgery, paediatrics, pathology, orthopaedics and family medicine [1–10].

Peer education after Topping can be defined as individuals from a similar social group who, as non-professional educators, help each other in learning and learn by teaching themselves [11]. Although there is no uniformly accepted definition of NPT, the already existing descriptions basically agree that it is a form of peer education in which a student in the teaching role participates in the same training but is at least one academic year ahead in his or her studies than the student in the student role [12, 13].

A widely accepted principle of the method is to exploit the benefits of cognitive and social congruence. Cognitive congruence (i.e., similar prior knowledge and study experiences) allows the use of language that is certain to be understood by the learner, as well as the appropriate choice of the logic and conceptual framework of the explanation, which helps the learner to understand. Social congruence (i.e., a similar role) allows for the creation of a calm and easy educational environment [13–15].

Although several publications have reported positive changes (such as the development of confidence or understanding) using the NPT method, these are mostly based on subjective impressions and opinions. Only a few studies were found that examined the effectiveness of NPT using objective and prospective techniques [1, 2, 16–18].

We have found just one publication by Preece et al. examining the effectiveness of NPT in teaching surgical skills. They found the use of peer-assisted learning as an effective, cheap and sustainable way to teach suturing skills, although they did not involve control group in their study [19].

## Methods

The aim of our research was to objectively investigate the effectiveness of NPT related to basic surgical skills.

### Main hypothesis

Involvement of NPT in surgical education improves the exam results of the students participating in the course.

### Secondary hypothesis

Involvement of NPT in education improves the satisfaction of students participating in the course.

### Study design

Single-blinded, prospective, parallel group, randomized controlled trial.

### Population of the study

Our sample size calculation was based on the test results of previous years. The study sample estimate envisaged the involvement of 58 students, estimating a 3-point difference between the study and control groups (α = 0.05, β = 0.1). Based on these, we included 60 volunteer students (30 women and 30 men, with a mean age of 22.6 ± 2.2 years) in the study. The criteria for student participation was the successful completion of the ‘Basics of Surgery’ course for the third-year pre-clinical curriculum of our university; however, the student should not complete the surgery summer practice after the fourth-year. So, participants were third- and fourth-year medical students.

Three volunteer peer educators were also involved in the research. The NPT tutors were sixth-year medical students who had successfully completed the surgical subjects included in our university’s compulsory curriculum, completed the simulation training centre’s own NPT preparation course (including an exam with test-training), and had a minimum of 1 year of NPT experience (NPT tutors).

The teaching staff consisted of three practicing clinical physicians working in the manual medical profession with at least 3 years of teaching experience (instructors).

Video recordings were evaluated with the involvement of three senior instructors who had a minimum of 10 years of teaching experience and 5 years of examination experience as clinical specialists working in the manual profession (senior instructors).

The study was approved by the Institutional Ethical Review Board (7719-PTE 2019).

### Protocol including procedural details

Prior to the course, all students underwent a preliminary aptitude test. In doing so, we assessed their existing skills in surgical knotting techniques, basic suture techniques, and basic laparoscopic skills. Knotting and suturing exercises were recorded on video, which was evaluated anonymously by the three senior instructors. We took into account the average of the scores given by the instructors. Laparoscopic skills were scored based on an evaluation sheet issued by the simulator.

1. Based on the scores obtained we divided the students into 4 groups (from poor to excellent performance). After this we have assigned a number between 1 and 6 randomly to the students within each group separately. Thereby six homogenous group of 10 with almost equal initial scores were created.
2. An NPT tutor and instructor were assigned to the three groups by drawing lots (study group), and then an instructor was assigned to the remaining three groups by drawing lots (control group). So, each instructor had a group where a NPT tutor would help with their work and one where they would hold the lesson alone.
3. All groups took a 20 × 45 min course with the same timeline on different weeks, including:
  - Deepening the knowledge of the instruments and introduction of suture materials – 45 min
  - Two-handed (surgical) and one-handed (Viennese) knotting - 4 × 45 min
  - Simple knot stitch, instrumental (apodactyl and atraumatic) knot and Zagreb’s knot – 2 × 45 min
  - Vertical mattress (Donati) and Allgöwer-Donati sutures - 2 × 45 min
  - Intracutaneous running sutures - 3 × 45 min
  - Laparoscopic basic (device knowledge and camera use) – 2 × 45 min
  - Laparoscopic skill development - 4 × 45 min
  - Free practice according to the student’s preference – 2 × 45 minutes
4. The skills of the students were assessed on the day after the end of the course in the same way as before the course.
5. At the end of the course, students completed a self-administered online anonymous questionnaire that measured their opinion about the course and the application of the NPT on a Likert scale of 5.

### Protocol of the evaluation

**Fig. 1:**
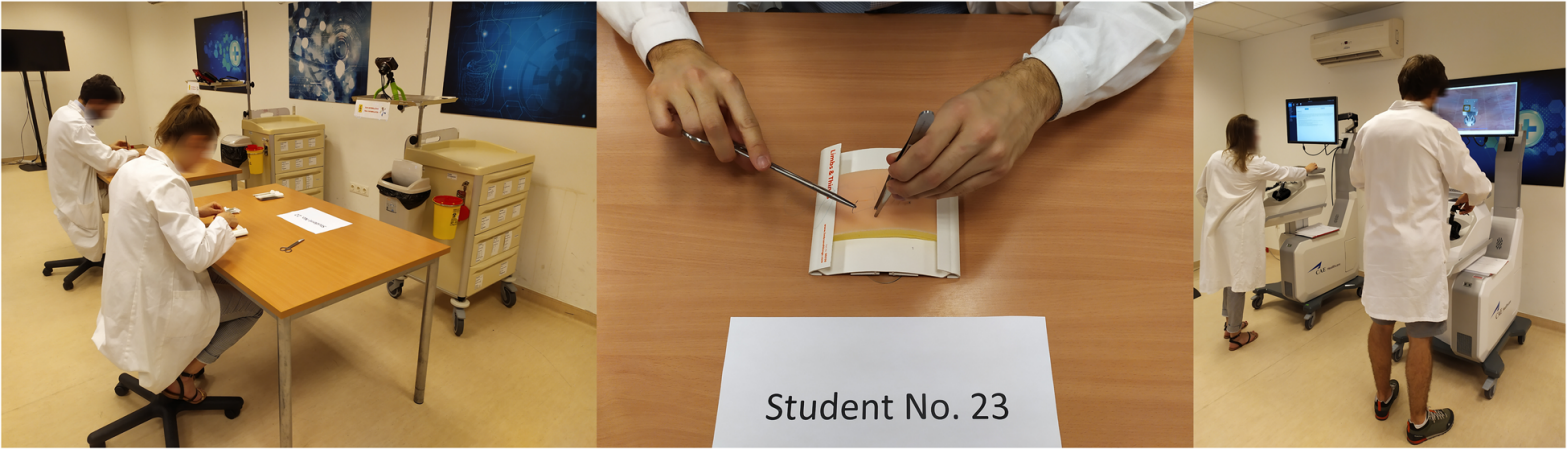
Post-course exam; left – recording of the suturing practices; middle – screenshot from the record; right – laparoscopic skills practices

Surveys were conducted before and on the day after the course under the supervision of the instructors. Students completed the assignments independently, without assistance, which were videotaped so that the student’s identity could not be identified (Fig. 1).

Videos were evaluated by senior instructors based on the Objective Structured Assessment of Technical Skill (OSATS) after they were settled and randomly ordered. To evaluate knotting, we used a slightly modified version of the criteria published by Shen et al. (Tables 1 and 2), and we developed our own criteria system to evaluate the sutures (Table 3) [20]. Prior to the course, we took test recordings with the help of the NPT tutors and instructors to check the usability of the scorecard, and the evaluators learned how to use the method. Training was continued until the senior instructors received an α value above 0.95 in intraclass correlation studies.

**Table 1:** Assessment of two-handed knot-tying techniques

	**1 point**	**2 points**	**3 points**
**Tension of knot-tying cords during the method**	Mostly loose	Mostly tight	Always tight
**Motion**	Many unnecessary moves	Less unnecessary movement	No unnecessary movement
**Direction of the first knot**	Wrong (knot failure)	Not ideal (knot failure)	Good
**Direction of knots**	Always the same	Partially alternating	Alternating
**Positioning of the knot**	Did not push down	Mostly pushed down	Always pushed down
**Respect of tissue**	The latex rubber band always moved in the direction of the hand (knotting material is too tight)	Unnecessary movements of trainer’s latex rubber band	The latex rubber band did not move towards the hand
**Stability of the knot**	Loose loop, the edges of the rubber did not line up	The edges of the rubber just fit together	Good stability, the edges of the rubber fit together
**Time**	60 s<	45-60 s	< 45 s

**Table 2:** Assessment of one-handed knot-tying techniques

	**1 point**	**2 points**	**3 points**
**Tension of knot-tying cords during the method**	Mostly loose	Mostly tight	Always tight
**Motion**	Many unnecessary moves	Less unnecessary movement	No unnecessary movement
**Direction of the first knot**	Wrong (knot failure)	Not ideal (knot failure)	Excellent
**Direction of knots**	Always the same	Partially alternating	Alternating
**Positioning of the knot**	Did not push down	Mostly pushed down	Always pushed down
**Respect of tissue**	The magnetic hook elevated	The magnetic hook elevated slightly	The magnetic hook did not elevate
**Stability of the knot**	Loose loop, fell off the hook	The knot slipped off the hook	Good stability, knot did not slip off the hook
**Time**	60 s<	45-60 s	< 45 s

**Table 3:** Score system used for assessment of sutures

	**1**	**2**	**3**
**Hold the needle at 1/3**	Not even once	Sometimes	Always
**90 ° between the surface and the needle**	Not even once	Sometimes	Always
**Appropriate usage of forceps**	Wrong	Intermediate	Excellent
**Appropriate direction and technique of knotting**	Wrong	Intermediate	Excellent
**Appropriate tightness of the knot**	Wrong	Intermediate	Excellent
**Appropriate position the skin edges**	Wrong	Intermediate	Excellent
**Appropriate distance from the skin edge**	Wrong	Intermediate	Excellent
**Appropriate distance between sutures**	Wrong	Intermediate	Excellent
**Respect of tissue**	Unnecessarily damaged the tissue	Handled the tissue carefully, but accidentally caused inadvertent damage	Did not damage the tissue
**Time and motion**	Did not finish within 4 min	Done in time, but a lot of unnecessary movements	Done in time, worked effectively
**Maintenance of asepsis**	Did not reach	Strived for it, but accidentally violated it	Reached
**SUM**			

#### Tasks of exam

1. Two-handed knot in the tension tissue model, with the latex rubber band of knotting trainer (three pieces with the right hand and three pieces with the left hand) evaluation according to OSATS (Table 1)
2. One-handed knot in the torn fabric model, with the magnetic hook of knotting trainer (three pieces with the right hand and three pieces with the left hand) evaluation according to OSATS (Table 2)
3. Simple interrupted suture, with atraumatic suturing material and instrumental knot with suture pad (three pieces) evaluation according to OSATS (Table 3)
4. Vertical mattress (Donati) suture, with atraumatic suturing material and instrumental knot with suture pad (three pieces) evaluation according to OSATS (Table 3)
5. Intracutaneous running suture, with atraumatic suturing material and instrumental knot with suture pad (4 cm) evaluation according to OSATS (Table 3)
6. Performing a “Peg transfer-Level 1” task on a LapVR v4.0.399.46816 (CAE Healthcare, Sarasota, FL, USA) simulator (the better result of two attempts) evaluation based on the evaluation sheet issued by the tool. If all the conditions were met, then the time allotted for the task was scored (one-tenth of the total time required to complete the task was subtracted from 35).

Statistical analysis was performed using the Mann-Whitney U Test and Wilcoxon signed- rank tests (IBM SPSS v23, IBM Corp., Armonk, NY, USA). The RANDBETWEEN function of Microsoft Excel v14.0.6112.5000 (Microsoft Corp., Redmond, WA, USA) was used for randomization. *P* <  0.05 was considered significant.

## Results

The results of the pre-course test in the control groups without the NPT tutor (the overall results of the students were 120.406 points) did not differ significantly from the study groups that included the NPT tutor (119.320 points, *p* = 0.679).

Taking into account the overall results of the students, a significant improvement was achieved in all areas during the completion of the course (average 33.690 point improvement, *p* <  0.001) (Table 4).

**Table 4:** Aggregate results of students’ surgical skills pre- and post-course

	**Pre-course**	**Post-course**	**Improvement**	**Wilcoxon (p)**	**Z**
**Overall results**	119.863	153.553	33.690*	< 0.001	−6.736
**Knotting**	27.767	39.783	12.017*	< 0.001	−6.740
Two-handed knotting	13.583	19.333	5.750*	< 0.001	−6.724
One-handed knotting	14.183	20.450	6.267*	< 0.001	−6.514
**Sutures**	72.533	88.217	15.683*	< 0.001	−6.618
Simple interrupted	24.067	29.467	5.400*	< 0.001	−6.205
Vertical mattress (Donati)	24.667	29.517	4.850*	< 0.001	−5.799
Intracutaneous running	23.800	29.233	5.433*	< 0.001	−6.342
**Laparoscopic basics**	19.563	25.553	5.990*	< 0.001	−6.283

The average points and the results of the Wilcoxon signed-rank test (*significant difference between pre- and post-course results)

In the groups where the NPT tutor assisted the teaching, students achieved a significantly greater improvement (30.180 vs. 37.200 point improvement, *p* = 0.036); however, this difference was significant only for the tasks related to knotting (9.300 vs. 14.733 point improvement, *p* = 0.003). There were no significant differences in tasks related to suturing (15.900 vs. 15.497 point improvement) and laparoscopic basics (4.980 vs. 7.000 point improvement) (Tables 5 and 6).

**Table 5:** The results of the pre- and post-course tests of the control and study groups

	**Pre-course**				**Post-course**				**Improvement**			
	**Control group**		**Study group**		**Control group**		**Study group**		**Control group**		**Study group**	
	**pt**	**%**	**pt**	**%**	**pt**	**%**	**pt**	**%**	**pt**	**%**	**pt**	**%**
**Overall results**	120,41	68,0%	119,32	67,4%	150,59	85,1%	156,52	88,4%	30,18	17,1%	37,20	21,0%
**Knotting**	28,73	59,9%	26,80	55,8%	38,03	79,2%	41,53	86,5%	9,30	19,4%	14,73	30,7%
Two-handed knotting	13,73	57,2%	13,43	56,0%	18,33	76,4%	20,33	84,7%	4,60	19,2%	6,90	28,8%
One-handed knotting	15,00	62,5%	13,37	55,7%	19,70	82,1%	21,20	88,3%	4,70	19,6%	7,83	32,6%
**Sutures**	71,37	72,1%	73,70	74,4%	87,27	88,2%	89,17	90,1%	15,90	16,1%	15,47	15,6%
Simple interrupted	23,77	72,0%	24,37	73,8%	29,33	88,9%	29,60	89,7%	5,57	16,9%	5,23	15,9%
Vertical mattress	24,63	74,6%	24,70	74,8%	29,07	88,1%	29,97	90,8%	4,43	13,4%	5,27	16,0%
Intracutaneous running	22,97	69,6%	24,63	74,6%	28,87	87,5%	29,60	89,7%	5,90	17,9%	4,97	15,1%
**Laparoscopic basics**	20,31	67,7%	18,82	62,7%	25,29	84,3%	25,82	86,1%	4,98	16,6%	7,00	23,3%

*pt* Point, *%* Percentage of the possible maximum points

**Table 6:** The changes between the pre- and post-course tests of the control and study groups

	**Control group**	**Study group**	**Difference**	**Mann-Whitney (p)**	**Z**
**Overall results**	30.180	37.200	7.020*	0.036	−2.100
**Knotting**	9.300	14.733	5.433*	0.003	−2.949
Two-handed knotting	4.600	6.900	2.300*	0.011	−2.536
One-handed knotting	4.700	7.833	3.133*	0.004	−2.849
**Sutures**	15.900	15.467	−0.433	0.865	−0.170
Simple interrupted	5.567	5.233	−0.333	0.789	−0.267
Vertical mattress (Donati)	4.433	5.267	0.833	0.528	−0.631
Intracutaneous running	5.900	4.967	−0.933	0.422	−0.802
**Laparoscopic basics**	4.980	7.000	2.020	0.156	−1.420

The average points and results of the Mann-Whitney U test (*significant difference between the control and study groups)

Based on the feedback of students, although students positively assessed the presence of demonstrators (4.80 on a Likert scale of 5), this did not significantly affect their satisfaction (4.77 vs. 4.83, *p* = 0.28) (Table 7).

**Table 7:** Results of students’ feedback

	Control group	Standard deviation	Study group	Standard deviation	Mann-Whitney (p)	Z
**1. Do you consider the material taught to be useful for your future profession?**	4.90	0.31	4.80	0.41	0.28	−1.08
**2. How do you judge the level of the course?**	4.50	0.68	4.43	0.73	0.74	−0.33
**3. Have you found the course interesting?**	4.70	0.47	4.60	0.62	0.66	−0.44
**4. Do you think the lessons were easy to understand?**	4.87	0.35	4.87	0.35	1.00	0.00
**5. Did you have enough time for practicing?**	4.97	0.18	4.83	0.38	0.09	−1.71
**6. Did the course leader/demonstrator pay enough attention to you?**	4.83	0.75	4.83	0.38	0.26	−1.13
**7. How do you feel and how much have your manual skills developed?**	4.67	0.55	4.47	0.63	0.18	−1.33
**8. All in all, how would you evaluate the course?**	4.83	0.46	4.77	0.50	0.51	−0.66
**9. Was the presence of the NPT tutor on the course useful?**			4.70	0.53		
**10. Could the NPT tutor help your progress?**			4.67	0.55		
**11. All in all, how would you judge the presence of the demonstrator?**			4.8	0.41		

Average result, standard deviation and results of the Mann-Whitney U Test

## Discussion

In the course of our research, we assessed the effect of the technical application of NPT on the development and satisfaction of students using an objective method among homogenized groups.

### Main hypothesis

We successfully proved our main hypothesis that the involvement of a peer educator in the teaching of basic surgical skills significantly improved the exam results of the students.

The course where also an NPT tutor attended the students’ education showed significantly better results in the tasks related to knotting, while no differences were found between the study and control groups in the tasks related to suturing and basic laparoscopic techniques. This controversial result may be due to several reason. The need for a high degree of personalized attention to the teaching of knotting can be one of them. By breaking it down into its elements and presenting professional hand movements individually, it is easier to learn the right technique. In suturing and laparoscopy, the appropriate technique and video assistance can also be effectively demonstrated for larger groups. At the same time, the instructor can check the end result more easily in larger groups, and the self-checking processes are easier as well. Viewing the finished seam line or the feedback provided by the laparoscopy simulator, it easily reveals type errors without the instructor. Another reason can be, that the post-course exam result of the suture skills reached the 88,2% of the possible maximum points in the control group leaving only a few potential for more improvement in the study group. We also need to acknowledge that 6 session of 45 min of laparoscopy education is probably a too short amount of training time for such complex skills to see the effect of NPT.

Although we did not find any publication in the literature, we reviewed the ones that we could directly compare to our results. They are consistent with our general findings that the application of NPT could improve students’ exam scores.

In the study by Preece et al., senior medical students delivered two suturing workshops to 35 students. They had significant improvement in the number of the completed number of sutures and inter-suture distance. All students found the workshop helpful and the teaching environment relaxed. Furthermore, 87% of the students reported that the workshop increased their interest in a surgical career [19].

In randomized controlled studies, Hudsen and Tokin demonstrated that NPT tutors can be at least as effective in teaching history and physical examination skills as graduate doctors, as well as others in the field of resuscitation training, bladder catheterization and intravenous cannulation [16–18].

In basic science, Gallan et al. (biochemistry), Jackson et al. (physiology), and Sawyer et al. (first-year curriculum) demonstrated the effectiveness of the use of additional NPT courses [21–23]. Alcamo et al. demonstrated the effectiveness of the USMLE Step 1 preparatory NPT course [24]. However, these studies are limited by the fact that NPT education was present as a complement, an additional learning opportunity.

In their retrospective study, Cate et al. compared the groups taught by NPT tutors and faculty for 36 courses (in the topic of circulation and metabolism) and found higher scores in 29 cases compared the groups taught by only NPT tutors [15]. The effectiveness of the method for teaching musculoskeletal competence has also been demonstrated with the involvement of orthopaedic residents [2].

In the reviewed literature, only Batchelder et al. did not find the application of the NPT method effective in terms of student exam scores, although they also reported that students in the programme felt more prepared and “more familiar with the style of exam questions” when there were NPT tutors [25].

### Secondary hypothesis

Our results were insufficient to judge possible change in student’s satisfaction since the overall satisfaction of the control group reached 4,83 on a scale of 5 leaving no room for more improvement. However, students, in line with the available international literature, found the application of the NPT technique useful and felt it helped their development [1, 3, 7].

## Limitations

Based on our result it can not be declared that the reason of the significant improvement is the NPT as method, and not just the involving of an extra tutor (“workforce”), although exploring the reasons was not aim of this study.

The senior instructor who evaluated the admissions was aware that she was looking at taking a pre- or post-course exam, which could affect her evaluation. However, the main issue of the present study was not to assess the overall improvement achieved during the course, but to compare the study and control groups, for which the evaluation was completely blind.

The personality of the demonstrator and instructor may have influenced the result. Although in the case of the instructor, we tried to eliminate this by the fact that all instructors also participated in the training of one control and one study group. At the same time, we tried to further reduce this effect by forming six groups and involving several instructors and demonstrators.

Cross-examination may be a more ideal choice to answer this question. But due to the nature of the curriculum, we thought this would be very difficult to do.

## Conclusions

We could confirm that the presence of a peer educator (NPT tutor) had a positive impact on student development.

Based on the results of our research, the application of the NPT technique can be a cost-effective and mutually beneficial way to increase the impact of basic surgical skills’ training.

## Data Availability

The datasets generated and/or analysed during the current study are not publicly available due data protection of the participants but are available from the corresponding author on reasonable request.

## Abbreviations

NPT: Neer-peer teaching
OSATS: Objective Structured Assessment of Technical Skill
SUM: Summary
pt.: Point

## References

[1] Shenoy A, Petersen KH (2020). Peer tutoring in preclinical medical education: a review of the literature. Med Sci Educ.

[2] Schiff A, Salazar D, Vetter C, Andre J, Pinzur M (2014). Results of a near-peer musculoskeletal medicine curriculum for senior medical students interested in orthopedic surgery. J Surg Educ.

[3] Rashid A, Chan SC, Choa G, Eboreime O (2019). Evaluating the effectiveness of using near-peer tutors in teaching first-year medical students. Future Healthc J.

[4] Alberti H, Rosenthal J, Kirtchuk L, Thampy H, Harrison M (2019). Near peer teaching in general practice: option or expectation?. Educ Prim Care.

[5] Musbahi A, Sharpe A, Straughan R, Ong S, Alhaddabi A, Reddy A (2019). A near-peer regional surgical teaching programme designed by medical students, delivered by junior doctors. Med Educ Online.

[6] Gottlieb Z, Epstein S, Richards J (2017). Near-peer teaching programme for medical students. Clin Teach.

[7] Karamaroudis S, Poulogiannopoulou E, Sotiropoulos MG, Kalantzis T, Johnson EO. Implementing change in neuroanatomy education: organization, evolution, and assessment of a near-peer teaching program in an undergraduate medical school in Greece. Anat Sci Educ. 2020. 10.1002/ase.1944. 10.1002/ase.1944 31955512

[8] Jenkinson A, Smith A, Doyle S, Gorman K, Murphy S (2019). Near peer teaching in a paediatric healthcare setting. Ir Med J.

[9] Tayler N, Hall S, Carr NJ, Stephens JR, Border S (2015). Near peer teaching in medical curricula: integrating student teachers in pathology tutorials. Med Educ Online.

[10] Burgess A, McGregor D, Mellis C (2014). Medical students as peer tutors: a systematic review. BMC Med Educ.

[11] Topping KJ (1996). The effectiveness of peer tutoring in further and higher education: a typology and review of the literature. High Educ.

[12] Bulte C, Betts A, Garner K, Durning S (2007). Student teaching: views of student near-peer teachers and learners. Med Teach.

[13] Cate OT, Druning S (2007). Dimensions and psychology of peer teaching in medical education. Med Teach.

[14] Cate OT, Druning S (2007). Peer teaching in medical education: twelve reasons to move from theory to practice. Med Teach.

[15] Cate OT, van de Vorst I, van den Broek S (2012). Academic achievement of students tutored by near-peers. Int J Med Educ.

[16] Tolsgaard MG, Gustafsson A, Rasmussen MB, Høiby P, Müller CG, Ringsted C (2007). Student teachers can be as good as associate professors in teaching clinical skills. Med Teach.

[17] Perkins GD, Hulme J, Bion JF (2002). Peer-led resuscitation training for healthcare students: a randomised controlled study. Intensive Care Med.

[18] Hudson JN, Tonkin AL (2008). Clinical skills education: outcomes of relationships between junior medical students, senior peers and simulated patients. Med Educ.

[19] Preece R, Dickinson EC, Sherif M (2015). Peer-assisted teaching of basic surgical skills. Med Educ Online.

[20] Shen Z, Yang F, Gao P (2018). A novel clinical-simulated suture education for basic surgical skill: suture on the biological tissue fixed on standardized patient evaluated with Objective Structured Assessment of Technical Skill (OSATS) tools. Invest Surg.

[21] Gallan AJ, Offner GD, Symes K (2016). Vertical integration of biochemistry and clinical medicine using a near-peer learning model. Biochem Mol Biol Educ.

[22] Jackson TA, Evans DJ (2012). Can medical students teach? A near-peer-led teaching program for year 1 students. Adv Physiol Educ.

[23] Sawyer SJ, Sylvestre PB, Girard RA, Snow MH (1996). Effects of supplemental instruction on mean test scores and failure rates in medical school courses. Acad Med.

[24] Alcamo AM, Davids AR, Way DP, Lynn DJ, Vandre DD (2010). The impact of a peer-designed and -led USMLE Step 1 review course: improvement in preparation and scores. Acad Med.

[25] Batchelder AJ, Rodrigues CM, Lin LY, Hickey PM, Johnson C, Elias JE (2010). The role of students as teachers: four years’ experience of a large-scale, peer-led programme. Med Teach.

[26] Schlégl ÁT, Pintér Z, Kovács A, et al. Teaching basic surgical skills using homemade tools in response to COVID-19. Acad Med. 2020. 10.1097/ACM.0000000000003586. 10.1097/ACM.0000000000003586 PMC7363365 32657784

